# Increasing Resistance of Coagulase-Negative Staphylococci in Total Hip Arthroplasty Infections: 278 THA-Revisions due to Infection Reported to the Norwegian Arthroplasty Register from 1993 to 2007

**DOI:** 10.1155/2014/580359

**Published:** 2014-10-09

**Authors:** Olav Lutro, Håkon Langvatn, Håvard Dale, Johannes Cornelis Schrama, Geir Hallan, Birgitte Espehaug, Haakon Sjursen, Lars B. Engesæter

**Affiliations:** ^1^Department of Medicine, Haukeland University Hospital, 5021 Bergen, Norway; ^2^Department of Clinical Science, University of Bergen, 5018 Bergen, Norway; ^3^Department of Orthopaedic Surgery, Haukeland University Hospital, 5021 Bergen, Norway; ^4^The Norwegian Arthroplasty Register, Department of Orthopaedic Surgery, Haukeland University Hospital, 5021 Bergen, Norway; ^5^Department of Clinical Medicine, University of Bergen, 5018 Bergen, Norway

## Abstract

We investigated bacterial findings from intraoperative tissue samples taken during revision due to infection after total hip arthroplasty (THA). The aim was to investigate whether the susceptibility patterns changed during the period from 1993 through 2007. Reported revisions due to infection in the Norwegian Arthroplasty Register (NAR) were identified, and 10 representative hospitals in Norway were visited. All relevant information on patients reported to the NAR for a revision due to infection, including bacteriological findings, was collected from the medical records. A total of 278 revision surgeries with bacterial growth in more than 2 samples were identified and included. Differences between three 5-year time periods were tested by the chi-square test for linear trend. The most frequent isolates were coagulase-negative staphylococci (CoNS) (41%, 113/278) and *Staphylococcus aureus* (19%, 53/278). The proportion of CoNS resistant to the methicillin-group increased from 57% (16/28) in the first period, 1993–1997, to 84% (52/62) in the last period, 2003–2007 (*P* = 0.003). There was also significant increase in resistance for CoNS to cotrimoxazole, quinolones, clindamycin, and macrolides. All *S. aureus* isolates were sensitive to both the methicillin-group and the aminoglycosides. For the other bacteria identified no changes in susceptibility patterns were found.

## 1. Introduction

The development of bacterial resistance has been an emerging problem since the introduction of the first antibiotics [1]. Studies of prosthetic joint infections (PJIs) have shown a high prevalence of methicillin-resistant* Staphylococcus aureus *(MRSA), methicillin-resistant* Staphylococcus epidermidis* (MRSE), extended-spectrum beta-lactamase-resistant Gram-negative bacteria (ESBL), and other highly resistant bacteria in PJI [2, 3].

Norway has a lower incidence of highly resistant bacteria compared to most European countries, but in selected populations, such as intensive care unit patients with infected foreign bodies (e.g., central venous catheters), an increasing proportion of infections are caused by MRSE [4, 5]. PJIs, such as an infected total hip arthroplasty (THA), impose a burden to the affected patients and are difficult to treat. An increased risk of revision due to deep infection after THA has been found in Norway as in the other Nordic countries [6, 7]. It has been suggested that increased bacterial resistance may have contributed to the increased risk of revision due to infection [8].

The aim in the present study was to investigate whether the susceptibility patterns had changed during the observation period from 1993 through 2007 for the bacteria causing deep infection after THA in Norway.

## 2. Material and Methods

Since its inception in 1987, The Norwegian Arthroplasty Register (NAR) has collected individual information on primary and revision THA [9]. Based on preoperative clinical examinations, laboratory tests, and intraoperative findings, the reason for revision is reported immediately after surgery by the surgeon to the NAR. The patients in this study were reported as revisions for infections. Bacterial findings were not reported to the NAR.

Patients from the period January 1, 1993, to September 30, 2007, were included. The study period was divided into three 5-year periods, which were compared to evaluate possible changes of resistance during the study period.

Based on registrations in the NAR, the ten hospitals with most THA-revisions due to infection, spread all over Norway, were visited by a single observer. This reflects large volumes of surgery, not higher rates of infection. Revision was defined as exchange or removal of parts or the whole prosthesis.

**Figure 1:**
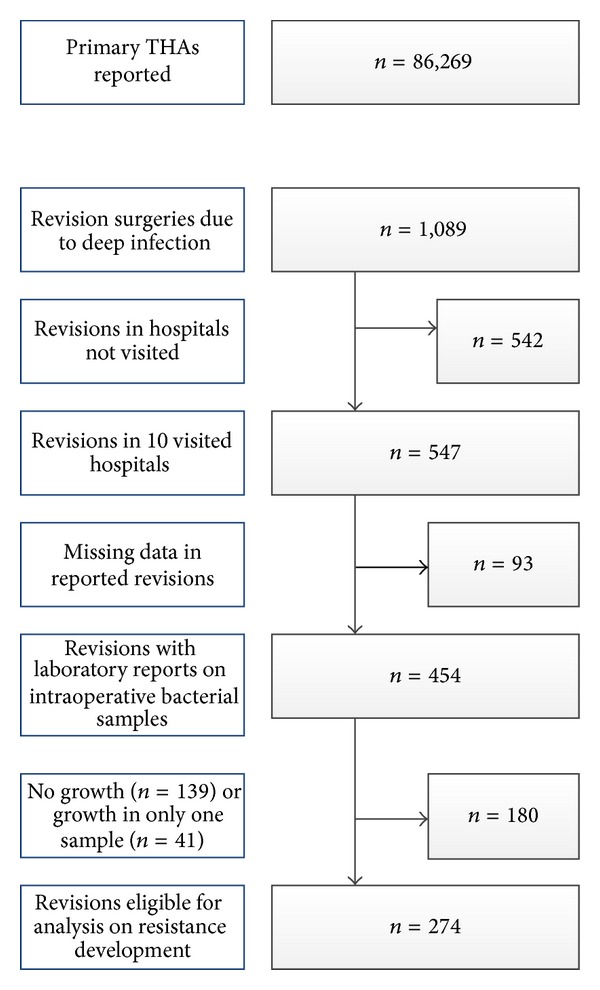
Flowchart showing the selection of patients.

For capacity reasons, we had to limit the number of visited hospitals to ten. These hospitals reported half of the revisions for infected THAs in the study period. Bacterial findings and susceptibility charts from the medical records were collected (Figure 1). To be included, the clinical diagnosis of infection had to be verified by two or more intraoperative tissue samples with valid growth of the same bacteria. Identification of the bacteria also had to include susceptibility panels. The tissue samples were handled fresh; mostly five samples were taken. There was no sonicated prosthesis included, as this method was, and still is not, in routine use in Norway. Thus, revisions reported as infections, but with no growth in intraoperative tissue samples, were not included (*n* = 139). These were mostly cases in which the patients had received antibiotics prior to surgery.

278 patients met the inclusion criteria. Patient characteristics are described in Table 1.

In addition, a written survey from all Norwegian microbiology laboratories was performed, asking about their culture techniques and growth media, incubation time, susceptibility panels, and breakpoints. In 2007, all the microbiology laboratories followed the recommendations for susceptibility patterns and breakpoints determining S, I, and R (SIR = Sensitive, Intermediate, Resistant) for the different bacteria, as recommended by the AFA (the Norwegian workgroup for questions regarding antibiotics). However, during the study period, the laboratories changed the susceptibility panels from 1, 2, 3, and 4 to SIR. We transformed 1 as S, 2 and 3 as I, and 4 as R. SIR was dichotomized into either S or R, regarding I as R to separate the sensitive bacteria from the rest.

The laboratories had, to some extent, used different methods, susceptibility panels, and incubation times over the 15 years studied. The mean incubation period was 7 days in 2007.

In general, few of the cultures were tested against linezolid, carbapenems, and rifampicin because those antibiotics were not part of the standard susceptibility panels used in Norway during the study period. In addition, few staphylococci were tested against the quinolones because the Norwegian regulatory authorities do not want the quinolones available in Norway (ciprofloxacin and ofloxacin) to be used routinely in the treatment of Gram-positive infections, in order to avoid the development of resistance.

We chose to combine methicillin, oxacillin, and cloxacillin in one group called the methicillin group. The laboratories used one of the above as a marker for resistance towards cloxacillin, dicloxacillin, and all cephalosporins. Furthermore, we combined the aminoglycosides gentamicin, tobramycin, and netilmicin into one aminoglycoside group. Ciprofloxacin and ofloxacin were combined in the quinolone group. Imipenem and meropenem were combined in the carbapenem group. Ceftazidime, ceftriaxone, and cefotaxime were combined in the third generation cephalosporin group.

The study was approved by the Regional Committee for Medical Research Ethics (number 2009/856b).

The chi-square test for linear trend was used to evaluate changes over time in the distribution of resistance. *P* values less than 0.05 were considered significant. Statistical analyses were performed using SPSS version 20 (SPSS Inc., 2004).

## 3. Results

The distribution of bacteria isolates is presented in Table 2. Coagulase-negative staphylococci (CoNS) were the dominating bacteria (41%), followed by* S. aureus *(19%). The results for antibiotic susceptibility are summarized in Table 3.

### 3.1. Coagulase-Negative Staphylococci

We found a high proportion of resistant strains among CoNS. Resistance increased with time. All CoNS cultures retained full susceptibility only to linezolid (only tested the last years) and vancomycin.

**Figure 2:**
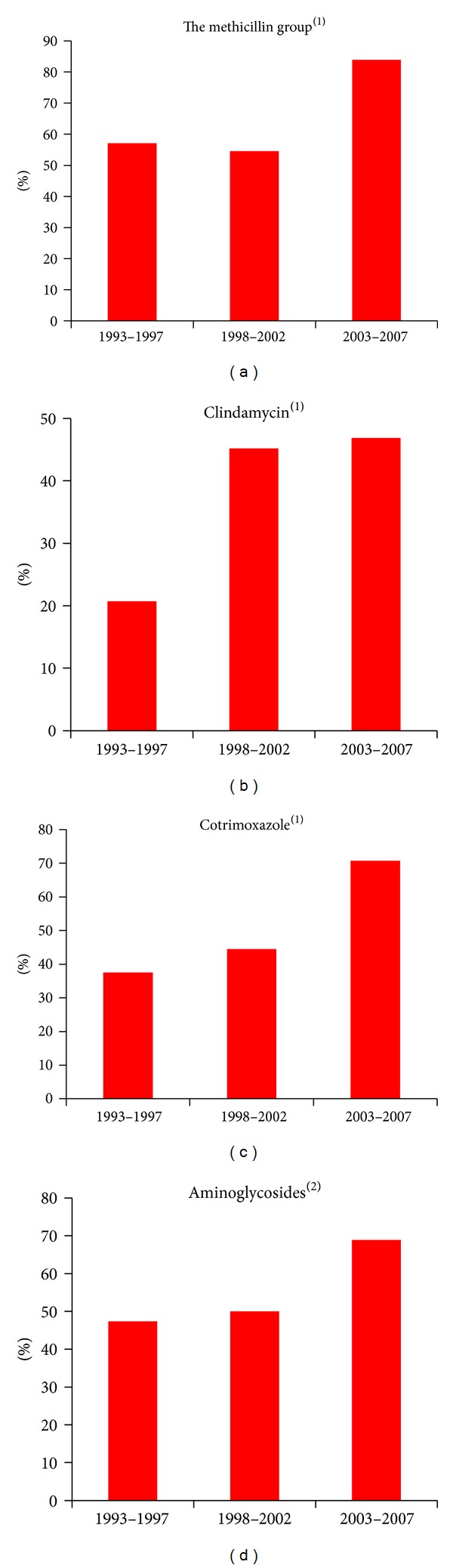
Development of resistance in coagulase-negative staphylococci towards selected antibiotics. Percent resistance is present on the *y*-axis, time period on the *x*-axis. (1) Significant increase in resistance for methicillin, clindamycin, and cotrimoxazole (*P* < 0.05). (2) Not significant increase in resistance for aminoglycosides (*P* = 0.15).

Resistance significantly increased over time to the methicillin group (*P* = 0.003), clindamycin (*P* = 0.048), trimethoprim/sulfamethoxazole (cotrimoxazole) (*P* = 0.03), quinolones (*P* = 0.03), and macrolides (*P* = 0.03). A trend of increased resistance was seen for aminoglycosides (*P* = 0.15) (Figure 2). Only a few rifampicin-resistant strains were identified, and only during the last 5-year period.

### 3.2. *Staphylococcus aureus*

All* S. aureus* cultures were susceptible to aminoglycosides, the methicillin group, rifampicin, vancomycin, linezolid, and cotrimoxazole. A few strains were found to be resistant to fusidic acid, clindamycin, quinolones, and macrolides.

### 3.3. Streptococci

All streptococci were susceptible to penicillin. A few strains were found to be resistant to clindamycin and macrolides.

### 3.4. Enterococci

All enterococci were susceptible to linezolid and vancomycin, and only one was resistant to ampicillin. However, a large proportion of the enterococci were resistant to aminoglycosides throughout the study period. We did not have information on whether some of the enterococci were highly resistant, so-called high-level gentamicin-resistant enterococci (HLGRE).

### 3.5. Gram-Negative Bacteria

The Gram-negative bacteria were all but one susceptible to aminoglycosides. A few strains were resistant to quinolones; a large proportion of the strains were resistant to ampicillin.

## 4. Discussion

We found a high proportion of resistant strains among CoNS, and we found that resistance increased with time.

For many years, CoNS were considered incapable of causing serious clinical infection and discarded as contamination when found in periprosthetic tissue cultures. However, CoNS are now considered a major cause of PJI [8, 10]. We found that CoNS was the most frequent bacteria causing infected THA in Norway.

The CoNS are skin commensals. When found in patients outside hospital settings, these bacteria exhibit less antimicrobial resistance than bacteria isolated from hospitalized patients and hospital personnel. Rapid transformation from susceptible to resistant strains has been shown soon after patients have been hospitalized [11–13]. Pre- and postoperative hospitalization for primary THA may have influenced the finding of a high proportion of multidrug resistant CoNS, as could the extensive use of cement containing antibiotics.

We do not know if the increased proportion of resistant CoNS is due to a general transformation of the bacterial flora, or if it only reflects a selection of bacteria causing THA infection. Epidemiologic surveys of CoNS susceptibility are absent in Norway.

The emergence of drug resistance in CoNS has been shown to reflect the consumption of antibiotics [14, 15]. We do not have data on the use of antibiotics for each individual patient, except for prophylaxis in primary THA as reported to the NAR. The Norwegian Institute of Public Health has found an increase in the use of both cephalosporins and quinolones in Norway during our study period [16]. This may also have contributed to the increased resistance of CoNS found in infected THA.

Other studies of PJI in hip- and knee arthroplasty have found the proportion of MRSE among bacterial infections to be 62–72% [2, 3, 17, 18]. Our findings of 70% MRSE are similar to these findings.

Dale et al. found a 3-fold increased risk of revision due to THA infection during the time period 2003–2007 compared to the time period 1987–1992 [6]. In an editorial comment to Dale's paper, Walenkamp raised the question of whether increased bacterial resistance could be part of the explanation [8]. Our findings support Walenkamp's opinion that increased prevalence of MRSE could be a part of the explanation.

We found a high proportion of CoNS and enterococci resistant to the aminoglycosides. A trend towards increasing aminoglycoside resistance was found with CoNS, whereas the resistance was more or less unchanged for the other bacteria. Interestingly, Fulkerson found a higher rate of susceptible CoNS for aminoglycosides in a cohort from New York and Chicago (87%) compared to our Norwegian patients (51%) [19]. The explanation for this difference could be that in Norway gentamicin-loaded bone cement was used in most primary THAs. In contrast, in the United States cementless implants are predominant, and when bone cement is used in primary surgery, it mostly does not contain antibiotics [20].

We found no methicillin-resistant* S. aureus* (MRSA). In Norway, there is a very low incidence of MRSA compared to most other countries [4]. In an English study, an MRSA prevalence of 8% was found. In a study from Australia, a prevalence of 11% MRSA was found (6/53) [2, 3]. In a Swedish study of infected knee implants only 1/84 of the causal bacteria were MRSA, reflecting the low incidence of MRSA also in another Scandinavian country [17]. The favorable resistance patterns of* S. aureus* are also reflected by the lack of resistance to aminoglycosides, linezolid, rifampicin, cotrimoxazole, and quinolones, and only sporadic cases of resistance to macrolides, clindamycin, and fusidic acid.

The incidence of methicillin resistance is higher for CoNS than* S. aureus*. In Norway, strict measures have been taken to prevent the spread of MRSA, similar to the Netherlands, and these programs have been successful thus far [21]. Preventing the spread of MRSE has proven more difficult [22]. 

*Limitations*. The present study has some limitations. It is a retrospective study based on data from a national registry.

However, our data on revisions due to infection after THA were prospective, and the NAR has been found to have good completeness [23, 24]. Since we used the NAR to identify cases of infection for the purpose of collecting bacteriological data, and the hospitals were different in types and spread all over the country, we expect the selection bias to be minor. Hence, we assume that our findings are representative for the susceptibility patterns with bacteria causing infected THAs in Norway over the study period.

The diagnosis of infection was based on perioperative assessment by the orthopaedic surgeon, before culturing results of intraoperative tissue samples were available. Since only cases with growth of the same bacteria in two or more tissue samples were included, all revisions included in the present study should be true PJIs. However, these strict criteria led to a high amount of reported revisions disqualified due to no growth or only one positive sample. We did not include preoperative joint fluid collection, as we first and foremost wanted our included revisions to be true PJIs, and we wanted full susceptibility charts. Also, PJIs treated with debridement without change of liner or head, or antibiotic suppression therapy alone, were not reported to the NAR and thus not included in the present study.

*Clinical Implications*. In Norway, the common practice is to use cephalothin as systemic prophylaxis during surgery and gentamicin in bone cement as local prophylaxis for cemented THAs [25]. The most common empirical antibiotic therapy for suspected PJI has been a combination of cloxacillin and gentamicin. With 84% methicillin resistance and 67% aminoglycoside resistance for CoNS during the last time period, treatment failure could be the result of inadequate antibiotic coverage. Thus, preoperative sampling, such as aspiration or biopsy, is crucially important, especially in low-grade infections. Under these circumstances, the patients are normally nonseptic, and there is time to await the culture results before surgical and medical treatment of the infection. The second most common pathogen,* S. aureus*, is fully susceptible to both the prophylactic regimen and the empirical treatment.

The antibiotic treatment must be adjusted to the bacteriological findings. When MRSE is proven or likely the cause of infection, vancomycin should be added to bone cement or spacers if used in revision surgery and should also be part of the systemic treatment [26]. The newly published national guidelines for use of antibiotics in hospitals from The Norwegian Directorate of Health have now advocated the use of vancomycin as empirical treatment for PJI, partly based on data from our study [27].

## 5. Conclusion

We identified an increase in the proportion of PJI-causing methicillin-resistant CoNS over the study period. Adequate bacterial sampling is crucial for choosing the right antibiotic treatment. This is increasingly important given the emerging resistance of CoNS found in PJI in the present study.

## Figures and Tables

**Table 1:** Patient characteristics.

Variable	
Sex (%)	
Male	32,4
Female	67,6
Age (mean) (SD)	69,4 (10,9)
Diagnosis (%)	
Osteoarthritis	67,3
Inflammatory	4,0
Other	28,7
Antibiotic prophylaxis systemically (%)	
Yes	98,4
No	1,6
Method of fixation (%)	
Uncemented	11,1
Cement	
With antibiotics	72,2
Without antibiotics	16,7

**Table 2:** Type of bacteria.

	Frequency	Percent
Coagulase-negative staphylococci	113	41
*Staphylococcus aureus*	53	19
Streptococci	30	11
Enterococci	26	9
Gram-negative bacteria	17	6
Polymicrobial	27	10
Other microbes	12	4

Total	278	100

**Table 3:** Susceptibility to selected antibiotics, given as number of tested isolates susceptible to the given antibiotic/total number tested.

	CoNS			*S. aureus*			Enterococci			Gram-negative			Streptococci		
Time period (years)	93–97	98–02	03–07	93–97	98–02	03–07	93–97	98–02	03–07	93–97	98–02	03–07	93–97	98–02	03–07

Aminoglycosides	10/19	13/26	14/45	5/5	8/8	22/22	0/8	0/2	3/10	4/4	7/8	11/11			
Methicillin group	12/28	15/33	10/62	10/10	15/15	37/37									
Cephalosporins, 3rd generation										2/3	6/6	6/7			
Clindamycin	23/29	17/31	34/64	11/11	13/13	35/36							2/2	6/6	10/13
Quinolones	6/6	8/17	8/20	2/2	0/4	3/4				2/3	4/7	8/10			
Carbapenems										3/3	4/5	8/8			
Linezolid		3/3	38/38			11/11	1/1		10/10						
Rifampicin	4/4	3/3	22/26	1/1		6/6									
Fusidic acid	12/29	14/31	33/63	11/11	13/14	33/35									
Macrolides	16/20	9/22	29/57	7/7	11/12	32/33							3/3	5/6	8/11
Cotrimoxazole	10/16	10/18	12/41	4/4	7/7	24/24				3/3	5/7	9/9			
Ampicillin							9/9	3/4	20/20	1/4	4/9	1/10			
Penicillin													5/5	8/8	15/15
Vancomycin	26/26	23/23	51/51		8/8	7/7	10/10	6/6	3/3	15/15					

CoNS = coagulase-negative staphylococci.
